# Selectivity Enhancement in Multisensor Systems Using Flow Modulation Techniques

**DOI:** 10.3390/s8117369

**Published:** 2008-11-19

**Authors:** Noureddine El Barbri, Cristhian Duran, Jesús Brezmes, Nicolau Cañellas, José Luis Ramírez, Benachir Bouchikhi, Eduard Llobet

**Affiliations:** 1 Sensor Electronic & Instrumentation Group, Faculty of Sciences, Physics Department, Moulay Ismaïl University, Meekness, Morocco; E-Mails: elbarbri.noureddine@caramail.com (N. E. B.); benachir.bouchikhi@gmail.com (B. B.); 2 Department of Electronic Engineering, University of Pamplona, Pamplona, Colombia; E-Mail: cmduran@unipamplona.edu.co; 3 MINOS, Department of Electronic Engineering, University Rovira i Virgili, Tarragona, Spain; E-Mails: jesus.brezmes@urv.cat; nicolau.canyellas@urv.cat; joseluis.ramirez@urv.cat

**Keywords:** Metal oxide gas sensor, flow modulation, support vector machine, wavelet transform

## Abstract

In this paper, the use of a new technique to obtain transient sensor information is introduced and its usefulness to improve the selectivity of metal oxide gas sensors is discussed. The method is based on modulating the flow of the carrier gas that brings the species to be measured into the sensor chamber. In such a way, the analytes' concentration at the surface of the sensors is altered. As a result, reproducible patterns in the sensor response develop, which carry important information for helping the sensor system, not only to discriminate among the volatiles considered but also to semi-quantify them. This has been proved by extracting features from sensor dynamics using the discrete wavelet transform (DWT) and by building and validating support vector machine (SVM) classification models. The good results obtained (100% correct identification among 5 volatile compounds and nearly a 89% correct simultaneous identification and quantification of these volatiles), which clearly outperform those obtained when the steady-state response is used, prove the concept behind flow modulation.

## 1. Introduction

In the last ten years, considerable efforts have been made to use sensor dynamics as a source of multivariate information leading to an enhancement in the discrimination ability of poorly-selective metal oxide gas sensors. Hand-held ‘sniffers’ make use of simple sample delivery units based on pumps rather than mass-flow controllers. Because it is well known that sensor dynamics can be of help to increase the selectivity of metal oxide sensors, there is a need for developing uncomplicated methods to use transient information in such analysers. In fact, many authors have reported different strategies for modulating either the sensor operating temperature [1-8] or the analyte concentration [9-11].

The new method presented here consists of the application of a modulated control signal to the peristaltic pump of a sniffer, which results in the gas flow being modulated. The effect sought by applying flow modulation is as follows: the concentration of analytes at the surface of the sensor is modulated, which results in the sensors working in a cycled non-equilibrium regime where, for example, adsorption/desorption and reaction rates can be altered, leading to the development of specific response patterns. The method of flow modulation can be easily adapted to both static and dynamic headspace sampling strategies.

Here, as a proof-of-concept, we investigate whether it is possible to easily discriminate and quantify among five different vapours (benzene, toluene, methanol, *o*-xylene and *p*-xylene) over a broad concentration range using a flow-modulated sensor array. The method employed to extract features from the sensor transients is based on the discrete wavelet transform [8, 12] and the pattern recognition makes use of simple support vector machine algorithms [13-16].

## 2. Experimental

### 2.1 Set-up

**Figure 1.**
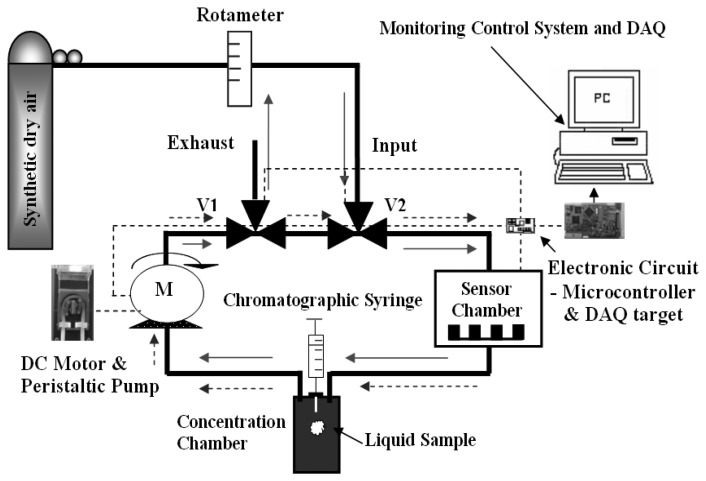
Experimental set-up during flow modulation experiments.

To achieve a flow-modulation capable sniffer, a closed loop system was designed which was based on a PC-controlled peristaltic pump and two three-way electro valves. Figure 1 shows the configuration devised. The stainless-steel sensor chamber housed three commercially-available Taguchi 8-series sensors (i.e. TGS-800,822 and 826). The system had two operating modes (cleaning phase and measurement phase). In the cleaning configuration, pure dry air enters the system through the first electro valve and cleans the peristaltic pump, the sensor chamber and the evaporation chamber. Solid arrows in Figure 1 mark the flow of clean air in this mode. In the measurement mode, air re-circulates in the closed circuit indicated by dashed arrows in Figure 1. Initially, the flow is kept constant at 250 sccm and a calculated quantity of a given pollutant in the liquid phase is sprayed into the evaporation chamber using a chromatographic syringe (since temperature, pressure and system volume were known, the volume of liquid to be injected could be calculated with the ideal gas theory [11]). This allows for obtaining the steady-state response of the sensors. Then, a square-wave flow modulation at a given frequency is initiated, which allows for measuring the transient response of the sensors. The system can generate other types of waveforms such as sinusoids or sawtooth functions, although they have not been used in this study.

A microcontroller commands the speed of the peristaltic pump, which directly translates into different flow rates. A PC programmed with a in-house-written program communicates with the microcontroller so that the user can select the flow rate and the frequency and type of waveform that is applied to modulate the flow. Via this program, the PC commands the microcontroller to open or close the electro valves and to change the configuration of the system depending upon the operating phase. Moreover, the PC records the sensor response (i.e. sensor conductivity) at the rate of 2 samples per second.

**Figure 2.**
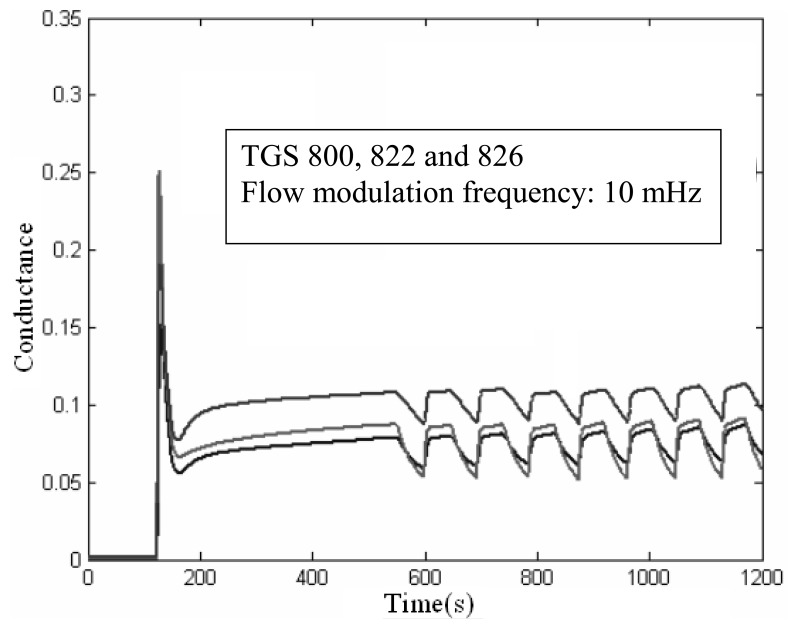
Dynamic response of three sensors. The flow modulation is applied from 550 s onwards.

Figure 2 shows that after a few modulation periods (e.g. 2) the transient pattern of the sensors becomes highly reproducible. In practice, a measurement would take 2 periods of the modulation frequency to complete.

Additionally, a temperature sensor was located inside the sensor chamber (placed perpendicular to the flow and between the chamber inlet and the gas sensors) so the effect of flow modulation in the temperature of the chamber (and hence in the operating temperature of the sensors) could be estimated. It was found that, no matter the flow modulation frequency applied, the temperature fluctuations inside the sensor chamber were never higher than ± 0.2°C. Such as small temperature variation confirms that the response patterns observed in Figure 2 are due to the effective modulation of analyte concentration and not to a periodic heating and cooling of the sensors.

### 2.2 Databases, Feature Extraction and Processing

In total, seven different databases where gathered, which corresponded to six flow modulation frequencies (i.e. 10, 20, 30, 40, 60 and 80 mHz) and an additional one that grouped measurements performed without modulating the flow (i.e. static measurements). Five different vapours (benzene, toluene, methanol, *o*-xylene and *p*-xylene) at three different concentrations (200, 400 and 2,000 ppm) were measured. Each measurement was replicated three3 times, which gave a total of 315 independent measurements. All this data were gathered in a disordered way during a period of two months.

The raw data consisted of the conductance change experienced by the sensors after the injection of a given species into the evaporation chamber and before a flow modulation was applied (case of static measurements), or in a period of the sensor conductance transient (case of flow modulation).

Different pre-processing strategies (e.g. mean-centring or auto scaling) were used to determine how much the mean amplitude, variance and waveform from each sensor response contributed to the correct identification of the species considered. Characteristic features from the sensor transient response were extracted by using the discrete wavelet transform. Pre-processed data were then used to build and validate support vector machine (SVM) classification models aimed at identifying the different species and also at determining their concentration. Since simple SVMs are for binary classification, multi category SVM classifiers were built using a one versus one approach [16, 17]. The feature extraction and pattern recognition techniques employed were implemented using standard toolboxes and functions from MATLAB®.

## 3. Results and Discussion

**Figure 3.**
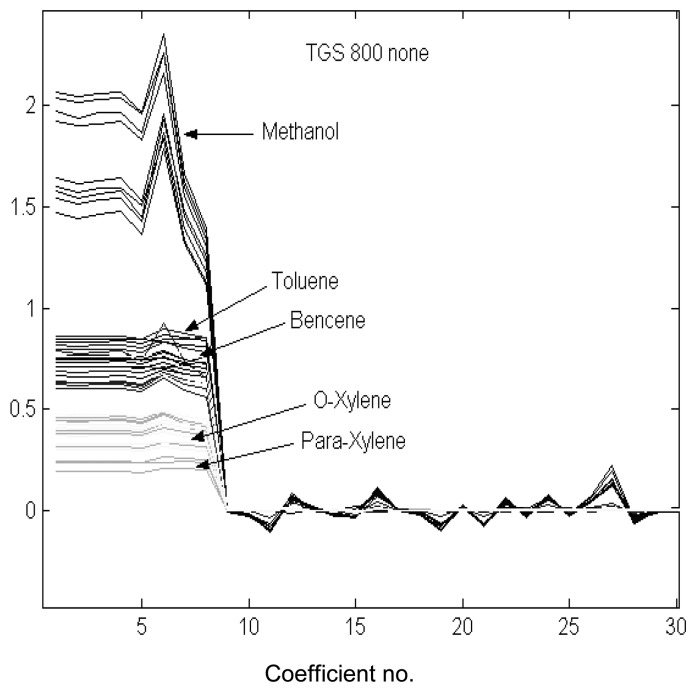
Wavelet coefficients of the DWT performed on the responses of sensor TGS 800. The flow modulation frequency was set to 10 mHz.

In order to perform the DWT of the pre-processed sensor transients, the fourth Daubechies function (db4) was used as the mother wavelet. This choice was based on our previous experience with temperature-modulated metal oxide gas sensors [8, 12]. The first eight wavelet coefficients of the fifth-order decomposition of the signals were retained for further processing. Figure 3 shows the results of the wavelet decomposition for the transient response of sensor TGS 800 when the flow modulating frequency was 10 mHz and no pre-processing was employed. The values of the first 8 wavelet coefficients for methanol, *o*-xylene and *p*-xylene appear well apart, suggesting that these species would be easily discriminated using this sensor. On the other hand, the coefficient values for benzene and toluene clearly overlap, which implies that these volatiles would be hard to discriminate.

### 3.1 Volatile Identification and Quantification Using the Steady-state Sensor Response

In the first step the discrimination of the different volatiles was attempted without modulating the flow. As stated above, the steady-state sensor response consisted of the conductance change. This database comprised 45 measurements (i.e. five volatiles × three concentrations × three replicate measurements). A leave one out cross-validation method was implemented as follows. A SVM classification model was built using 44 out of the 45 measurements available. The performance of the model was then evaluated using the measurement that had been left out. This procedure was performed 45 times using always a different validation measurement. The performance in classification of the SVM models built was computed as the average over the 45 tests.

A one-against-one strategy was used to build the SVM classifier. This method builds *n* (*n*−1)/2 classifiers (*n* = 5 in identification and *n* = 15 in the simultaneous identification and quantification) where each one is trained using input patterns from two classes. To classify a new pattern the results obtained by the *n* (*n*−1)/2 classifiers are combined in a simple voting scheme. In case of equal number of votes between different classes, the one with smaller index is selected. Therefore, 10 SVM models were built for identification purposes. The first SVM was trained with the training samples in class 1 (i.e. methanol) with positive labels and all other training samples with negative labels, and so on. Different kernel functions were tested such as linear, polynomial (2^nd^ degree) and radial basis. The best results were obtained when a 2^nd^ degree polynomial was used as kernel function. A measurement per category (selected at random within the replicate measurements available in each category) was used to determine the optimal value of the penalty parameter, C, which is useful when building models where overlapping between classes is present, using a leave-one-out cross-validation. The values of C can lie in the interval [0, ∞] and large values lead to a small number of training samples being misclassified. For example, when C tends to infinity, no misclassifications are allowed among training samples. More details about the use of SVM can be found in [16].

Two different approaches were studied. In the first one, classification models were built using the information of one sensor only. This allows for studying and comparing the resolving power of every sensor. In the second approach, models were built that combined information from the three sensors. The validation results for SVM-based volatile identification using the steady-state response of the sensors are shown in Table 1. Correct identification peaks at 87.1% when the information from the three sensors is combined and a mean centring pre-processing method is employed. Confusions occur between some benzene and toluene samples and also between some *o*-xylene and *p*-xylene samples. On the other hand, if only one sensor is used, the identification ability dramatically deteriorates and remains below 58% regardless the sensor and pre-processing technique considered.

Additionally, a semi-quantitative analysis was performed. This consisted of the simultaneous identification and quantification of the measurements. A 15 category classification was envisaged (i.e. five volatiles × three concentrations) and the methods implemented to build and validate the SVM models were analogous to the ones explained above. Table 2 summarizes the quantification results, which are rather poor since success rate remains below 54%.

### 3.2 Volatile Identification and Quantification Using Flow Modulation

Once more, in the first step the discrimination of the different volatiles was attempted using the flow modulation approach. As stated before, eight features per sensor (i.e. the first eight coefficients of the 5-level wavelet decomposition of the transient signals) were extracted. In total 45 measurements were performed for each modulation frequency investigated and, similarly to the steady-state case, SVM models for the identification of volatiles were built and validated (i.e, using the leave-one-out procedure described above). Table 3 summarises the identification results reached with the six flow modulation frequencies studied. Confusions occur between some benzene and toluene samples only. From this table, it can be derived that modulating the flow at 20 or 30 mHz results in the best discrimination results. The five volatiles can be perfectly identified by the SVM models when either a mean centring or auto scaling pre-processing is employed at 20 mHz. They can also be correctly discriminated when an auto scaling pre-processing is employed at 30 mHz. Since the value of parameter C is lower at 20 mHz than at 30 mHz, the SVM models are simpler for the former and, therefore, a flow modulation at 20 mHz seems the better choice for volatile discrimination.

In the second step a 15-category classification was attempted (i.e. the simultaneous identification and quantification of volatiles). Once again, a leave-one-out training and validation technique was implemented. Table 4 summarises the results of this semi-quantitative analysis. This table shows that the best classification rate, which reaches nearly 89%, is obtained when the pre-processing technique is auto scaling and the flow modulation frequency is set to 30 mHz. It is worth mentioning that at such modulation frequency, confusions do not occur between the different volatiles but between different concentrations of a given volatile. In other words, misclassified samples represent an erroneous estimation of the concentration but not an error in the identification of the volatile species.

When the number of measurements is low, the leave-one-out method tends to be an optimistically-biased estimator of the true success rate of a classifier. Therefore, a different validation approach was considered too. Since three replicate measurements were available per type of volatile and concentration, a three-fold validation strategy has been implemented. In this approach a training set is formed by selecting two of the three replicate measurements available and the third left out is in the training set. Three different folds (i.e. combinations) of training and validation sets can be defined and employed. The overall success rate in classification is then obtained as the average success rate on the three folds. This method was applied to estimate the success rate in vapour identification (i.e. 5-category classification) and in the simultaneous identification and quantification of vapours. The best results were obtained when the modulating frequency and the constant C were set to 30 mHz and 10, respectively.

A 100% success rate in vapour identification and an 82% success rate in the simultaneous identification and quantification were obtained. Keeping in mind that the 3-fold validation strategy tends to be a pessimistically-biased estimator of the true success rate of a classifier, it can be derived that flow modulation allows for a perfect identification and a fair quantification of the vapours studied.

Different factors may explain why 20 and 30 mHz are the flow modulation frequencies that lead to the best results in vapour identification and in the simultaneous identification and quantification, respectively. These frequencies seem appropriate for effectively modifying the concentration of analytes at the sensor surface, making the sensors to work in non-equilibrium regime. This helps specific patterns of sensor response to develop. In fact, adsorption, desorption and reaction kinetics are slow and too high a modulation frequency could not be appropriate for developing different sensor response patterns for each different volatile measured. Additionally, the volume of the sensor chamber and the tubing act as a low-pass filter for the flow modulation. These two reasons may explain why modulation frequencies of 40 mHz or higher lead to poorer results. Finally, if the frequency of the flow modulation is too low (i.e. 10 mHz), the sensors may work in quasi-equilibrium (i.e. similarly to when they are operated in steady-state) and the volatile identification results do not differ very much from those obtained measuring in a steady-state regime.

In summary, the use of transient response information via flow modulation is more advantageous than the use of the steady state sensor response, since the former clearly outperforms the latter both in the identification success rate (100% versus 87.1%) and in the semi-quantification success rate (89% versus 53.8%).

## 4. Conclusions

In this paper, we have introduced a new technique (i.e. flow modulation) to obtain transient information from a sensor array. By modulating the flow of the gas mixture to be measured, the analytes' concentration are altered. By doing so it was found that reproducible patterns in the sensor response developed, which carried important information for discriminating and quantifying the different volatiles considered.

Important information from the transient response of the sensors was extracted using the DWT and employed to build and validate SVM classification models. Additionally, similar SVM models were built using the steady-state response of the sensors. The use of flow modulation led to a 100% success rate in identification and a 89% or 82% success rate in the simultaneous identification and quantification of the volatiles (depending if a leave-one-out or a three-fold validation approach is implemented). These results compare very favourably to the ones obtained when the steady-state response of the sensors was considered only.

The fact that reliable flow modulation can be obtained using uncomplicated methods (e.g. a peristaltic pump) makes this option very interesting for simple gas analysis equipment such as hand-held devices or sniffers.

## Figures and Tables

**Table 1.** Success rate in vapour identification (%) using the steady state sensor response. Validation results are reported. The optimal value of parameter C is also shown.

**Pre-processing**	**TGS 800**	**TGS 822**	**TGS 826**	**All sensors**
None	54.2 C = 10	57.7 C = 10^2^	30.8 C = 10	82.3 C = 10
Mean-centring	49.9 C = 10^2^	57.7 C = 10^2^	29.2 C = 10	87.1 C = 10^3^
Auto scaling	53.3 C = 10^3^	57.7 C = 10^3^	23.1 C = 10^2^	84.4 C = 10^3^

**Table 2.** Success rate in the simultaneous identification and quantification of vapours (%) using the steady state sensor response. Validation results are reported. The optimal value of parameter C is also shown.

**Pre-processing**	**TGS 800**	**TGS 822**	**TGS 826**	**All sensors**
None	2.2 C = 10	8.6 C = 10	10.9 C = 10^3^	37.7 C = 10
Mean-centring	13.3 C = 10^3^	24.0 C = 10^3^	11.3 C = 10^3^	53.8 C = 10
Auto scaling	13.4 C = 10^3^	19.1 C = 10^3^	11.1 C = 10^4^	42.6 C = 10

**Table 3.** Success rate in vapour identification (%) using flow modulation. Validation results are reported. The optimal value of parameter C is also shown.

**Frequency**	**Pre-processing**	**TGS 800**	**TGS 822**	**TGS 826**	**All sensors**
10 mHz	None	86.7 C = 10	95.1 C = 10	97.7 C = 10	84.4 C = 10
	Mean-centring	79.2 C = 10^2^	88.3 C = 10	84.4 C = 10^3^	82.2 C = 10
	Auto scaling	82.2 C = 10	90.6 C = 10	90.6 C = 10^3^	88.9 C = 10

20 mHz	None	92.9 C = 10	97.0 C = 10	97.5 C = 10	99.7 C = 10
	Mean-centring	93.3 C = 10^2^	91.1 C = 10	97.8 C = 10^2^	100 C = 10
	Auto scaling	88.3 C = 10^4^	97.0 C = 10^2^	100 C = 10^2^	100 C = 10

30 mHz	None	91.1 C = 10	95.6 C = 10^2^	95.6 C = 10	97.8 C = 10
	Mean-centring	97.4 C = 10	95.9 C = 10^2^	99.7 C = 10^2^	100 C = 10^2^
	Auto scaling	95.6 C = 10^2^	88.6 C = 10^2^	95.4 C = 10^2^	97.8 C = 10^2^

40 mHz	None	90.6 C = 10	88.3 C = 10	79.2 C = 10	93.3 C = 10
	Mean-centring	74.7 C = 10	83.7 C = 10	83.1 C = 10^4^	95.6 C = 10
	Auto scaling	83.7 C = 10^4^	86.5 C = 10^4^	75.2 C = 10^2^	93.3 C = 10

60 mHz	None	83.1 C = 10	87.6 C = 10	92.8 C = 10	88.9 C = 10
	Mean-centring	78.3 C = 10^2^	73.8 C = 10^4^	85.3 C = 10^2^	84.4 C = 10^2^
	Auto scaling	72.4 C = 10^5^	76.8 C = 10^5^	79.2 C = 10^5^	84.4 C = 10^2^

80 mHz	None	70.2 C = 10	91.1 C = 10	83.7 C = 10^2^	97.7 C = 10
	Mean-centring	70.5 C = 10^2^	80.0 C = 10	84.4 C = 10^2^	95.5 C = 10
	Auto scaling	53.3 C = 10	88.9 C = 10^2^	78.1 C = 10^3^	97.7 C = 10

**Table 4.** Success rate in the simultaneous identification and quantification of vapours (%) using flow modulation. Validation results are reported. The optimal value of parameter C is also shown.

**Frequency**	**Pre-processing**	**TGS 800**	**TGS 822**	**TGS 826**	**All sensors**
10 mHz	None	64.5 C = 10	42.6 C = 10	61.8 C = 10	56.2 C = 10
	Mean-centring	60.9 C = 10	58.6 C = 10	60.0 C = 10	70.5 C = 10
	Auto scaling	68.9 C = 10^2^	62.2 C = 10^2^	51.4 C = 10^2^	59.8 C = 10

20 mHz	None	48.4 C = 10	55.0 C = 10	67.8 C = 10	45.2 C = 10
	Mean-centring	60.9 C = 10^2^	49.0 C = 10^2^	72.4 C = 10^2^	64.4 C = 10^2^
	Auto scaling	54.1 C = 10^2^	57.5 C = 10^2^	86.1 C = 10^2^	78.4 C = 10

30 mHz	None	72.4 C = 10	69.2 C = 10	81.6 C = 10	70.0 C = 10
	Mean-centring	73.8 C = 10	57.3 C = 10	72.4 C = 10^2^	77.8 C = 10
	Auto scaling	72.4 C = 10^2^	66.5 C = 10^2^	60.9 C = 10^2^	88.9 C =10

40 mHz	None	45.9 C = 10	66.8 C = 10	57.5 C = 10	50.5 C = 10
	Mean-centring	46.7 C = 10^2^	44.2 C = 10^2^	65.7 C = 10^2^	62.1 C = 10^2^
	Auto scaling	52.8 C = 10^2^	50.9 C = 10^2^	55.2 C = 10^2^	65.7 C = 10^2^

60 mHz	None	53.8 C = 10	65.7 C = 10	67.0 C = 10	56.5 C = 10
	Mean-centring	67.0 C = 10^2^	40.6 C = 10^2^	65.6 C = 10	65.6 C = 10^2^
	Auto scaling	44.2 C = 10^2^	44.2 C = 10^2^	59.8 C = 10^6^	72.4 C = 10

80 mHz	None	49.0 C = 10	67.8 C = 10	57.5 C = 10	70.1 C = 10
	Mean-centring	59.7 C = 10^2^	68.9 C = 10^2^	62.2 C = 10^2^	75.5 C = 10
	Auto scaling	44.3 C = 10	57.1 C = 10^2^	52.8 C = 10^2^	82.2 C = 10

## References

[1] Sears W.M., Colbow K., Consadori F. (1989). General characteristics of thermally cycled tin oxide gas sensors. Semicond. Sci. Technol..

[2] Sears W.M., Colbow K., Consadori F. (1989). Algorithms to improve the selectivity of thermally cycled tin oxide gas sensors. Sens. Actuat..

[3] Nakata S., Kaneda Y., Nakamura H., Yoshikawa K. (1991). Detection and quantification of CO gas based on the dynamic response of a ceramic sensor. Chem. Lett..

[4] Nakata S., Nakamura H., Yoshikawa K. (1992). New strategy for the development of a gas sensor based on the dynamic characteristics: principle and preliminary experiment. Sens. Actuat..

[5] Nakata S., Akakabe S., Nakasuji M., Yoshikawa K. (1996). Gas sensing based on a nonlinear response: discrimination between hydrocarbons and quantification of individual components in a gas mixture. Anal. Chem..

[6] Cavicchi R.E., Suehle J.S., Kreider K.G., Gaitan M., Chaparala P. (1995). Fast temperature programmed sensing for micro-hotplate gas sensors. Elect. Dev. Lett..

[7] Llobet E., Ionescu R., Al-Khalifa S., Brezmes J., Vilanova X., Correig X., Bârsan N., Gardner J.W. (2001). Multicomponent gas mixture analysis using a single tin oxide sensor and dynamic pattern recognition. IEEE Sens. J..

[8] Llobet E., Brezmes J., Ionescu R., Vilanova X., Al-Khalifa S., Gardner J.W., Bârsan N., Correig X. (2002). Wavelet transform fuzzy ARTMAP based pattern recognition for fast gas identification using a micro-hotplate gas sensor. Sensor. Actuat..

[9] Auerbach F. (1995). Pattern Recognition Using Gasmodulation. Techn. Dig. Transduc..

[10] Llobet E., Vilanova X., Brezmes J., Sueiras J.E., Correig X. (1998). Transient response of thick-film tin oxide gas sensors to multicomponent gas mixtures. Sensor. Actuat..

[11] Llobet E., Vilanova X., Brezmes J., Alcubilla R., Calderer J., Sueiras J.E., Correig X. (1997). Conductance-transient analysis of thick-film tin oxide gas sensors under successive gas-injection steps. Meas. Sci. Technol..

[12] Llobet E., Ionescu R. (2002). Wavelet Transform based fast feature extraction from temperature modulated semiconductor gas sensors. Sens. Actuat..

[13] Al-Khalifa S., Maldonado-Bascón S., Gardner J.W. (2003). Identification of CO and NO_2_ using a thermally resistive microsensor and support vector machine. IEEE Proc. Sci. Meas. Technol..

[14] Distante C., Ancona N., Siciliano P. (2003). Support vector machines for olfactory signals recognition. Sens. Actuat..

[15] Pardo M., Sberveglieri G. (2005). Classification of electronic nose data with support vector machines. Sens. Actuat..

[16] Gualdrón O., Brezmes J., Llobet E., Amari A., Vilanova X., Bouchikhi B., Correig X. (2007). Variable selection for support vector machine based multisensor systems. Sens. Actuat..

[17] Hsu C.W., Lin C.J. (2002). A comparison of methods for multiclass support vector machines. IEEE Trans. Neural Network..

